# Supplementary material for: Research on desiliconization of brown corundum fly dust and bauxite based on roasting-alkali leaching method
Source: PLoS One. 2025 Feb 5;20(2):e0316172. doi: 10.1371/journal.pone.0316172 (PMC11798484; doi:10.1371/journal.pone.0316172)
Supplement: Attachment — Submitted filename: Response to Reviewers.docx [file pone.0316172.s003.docx]

Dear Editor and Reviewers,

Thank you for forwarding the reviewers’ comments regarding our manuscript titled “Research on Desiliconization of Brown Corundum Fly Dust and Bauxite Based on Roasting-Alkali Leaching Method” PONE-D-24-27758 submitted to PLOS ONE. We have carefully considered each comment and have made the necessary adjustments to enhance the quality and clarity of our manuscript. Below, we address each point raised by the reviewers and describe the revisions made.

Reply to Reviewer #1

Comment 1: The keywords and detailed description of the methods with indication of technological parameters:

Response 1: We have added a section for keywords at the beginning of the manuscript to facilitate indexing and retrieval, and revised lines 48-64 to include detailed technological parameters such as temperature, reagent concentrations, process duration, and metals extraction degrees.

Comment 2: Table 1. Add the LOI content and Section 2.1. Add information about particle size distribution of the raw materials:

Response 2: We have updated Table 1 to include LOI content and the concentrations of CaO, MgO, Na_2_O and sulfur for the BCFD sample. The BCFD sample does not contain carbon and therefore not included in the table, We have tandardized the terminology throughout the manuscript, using “min” for minutes and “desilication” instead of “desiliconization”. It is a pity that the particle distribution of the raw material was not added, because due to the neglect of the previous work, it is inconvenient to make up for the work now, but we have added the pore size distribution before and after to illustrate the problem.

Comment 3: Add the information about chemical composition (g/L) after alkali leaching. Write the options for treatment this solution using the links. Add information about particle size distribution of the sample after desilication. What is the yield of the sample after roasting, and after desilication?

Response 3: We have included data on the chemical composition. We analyzed Figure 7 in the manuscript by adding the specific surface area and pore size distribution before and after desilication. Details regarding the yield after roasting and desilication have been added to the manuscript.

Comment 4: Figure 1. Add the chemical formulas of the minerals. Figure 3. Add the error bars to the columns and experimental points. Figure 3d, 7. Sign peak at 20 degrees.

Response 4: We have made the following data updates: Chemical formulas have been added to Figure 1. Error bars have been included in Figure 3 to represent variability. The peak at 20 degrees in Figures 3d and 7 is now labeled. Figure 8 has been recalculated and corrected as advised, ensuring the curves pass through point zero.

Comment 5: Authors should add the final flowsheet with the optimum technical parameters.

Response 5: The revised manuscript contains a detailed final flowchart with the best technical parameters, as shown in Fig 2.

Reply to Reviewer #2

Comment 1: Picture clarity is low, need to improve picture quality.

Response 1: All figures have been enhanced for clarity and resolution in the revised submission.

Comment 2: It is suggested that some expressions be modified,

Response 2: We have modified the expressions as suggested to improve readability and grammatical accuracy.

Comment 3: In line 69, the two sentences are not separated.

Response 3: We have corrected the cited grammatical errors and ensured proper sentence separation and punctuation throughout the manuscript.

Comment 4: There is a sign error in line 204.

Response 4: We have carefully revised this section and verified what may be present in the text.

Comment 5: Why choose 1050℃ for roasting activation temperature?

Response 5: Relevant literature shows that the activation temperature of silica in bauxite is in the range of 900-1100°C, and quartz will be formed if the temperature is too high, An explanation for selecting 1050°C as the roasting activation temperature has been added, including references to prior studies that validate this choice.

Comment 6: The desilication product XRD pattern suggests the addition of a standard card.

Response 6: The XRD pattern now includes a standard card for better comparison and validation of the phases identified.

We believe these revisions have addressed the concerns raised by the reviewers and have significantly improved the manuscript. We appreciate your consideration of our revised submission and eagerly await further feedback. Thank you for considering our responses, and we look forward to the potential acceptance and publication of our manuscripts.

Thank you for your time and attention.

Yours sincerely,

Nian Liu
