# Supplementary material for: Research on desiliconization of brown corundum fly dust and bauxite based on roasting-alkali leaching method
Source: PLoS One. 2025 Feb 5;20(2):e0316172. doi: 10.1371/journal.pone.0316172 (PMC11798484; doi:10.1371/journal.pone.0316172)
Supplement: Attachment — Submitted filename: Response_to_Reviewers_auresp_2.docx [file pone.0316172.s004.docx]

Dear Editor and Reviewers,

Thank you for forwarding the reviewers’ comments regarding our manuscript titled “Research on Desiliconization of Brown Corundum Fly Dust and Bauxite Based on Roasting-Alkali Leaching Method” PONE-D-24-27758 submitted to PLOS ONE. We carefully considered each review and made the necessary adjustments to improve the quality of our manuscripts. Below we have made changes to each of the points raised by the academic editors.

Comment1: Reviewers recommend that you cite specific work that has been previously published.

Response1: Because there is less literature on brown corundum fly dust and bauxite synergistic desiliconization, brown fused alumina is a more traditional industrial material, and there are few current studies, most of the studies on brown corundum fly dust are the extraction of gallium metal, among which there are few studies on synergistic desiliconization with bauxite, and the current popular research is cited in the manuscript. Citations 1 to 6 describe the characteristics of bauxite. Citations 7 to 10 describe the generation and characteristics of brown corundum fly dust, Citations 11 to 18 introduces the current research methods of bauxite, Some current research methods and results of bauxite are introduced, which provides a theoretical basis for our experiments, and Citations 19 to 21 introduces the research methods of brown corundum fly dust. Citations 22 to 30 describe the mechanistic issues that occur during the experiment. Due to the lack of articles related to the synergistic desiliconization of brown corundum fly dust and bauxite, the articles in related research fields are listed in this paper, and the experimental results show that the effect is better. Some citations have been added and removed from the manuscript. I hope my revisions are understood by the academic editors, sorry for the trouble.

Comment2: While revising your submission, please upload your figure files to the Preflight Analysis and Conversion Engine (PACE) digital diagnostic tool.

Response2: The figure files in this article have been uploaded to the Preflight Analysis and Conversion Engine (PACE) digital diagnostic tool, under the account ln15934767600@163.com.

We believe these revisions have addressed the concerns raised by the reviewers and have significantly improved the manuscript. We appreciate your consideration of our revised submission and eagerly await further feedback. Thank you for considering our responses, and we look forward to the potential acceptance and publication of our manuscripts.

Thank you for your time and attention.

Yours sincerely,

Nian Liu
